# Optineurin Negatively Regulates the Induction of IFNβ in Response to RNA Virus Infection

**DOI:** 10.1371/journal.ppat.1000778

**Published:** 2010-02-19

**Authors:** Jamel Mankouri, Rennos Fragkoudis, Kathryn H. Richards, Laura F. Wetherill, Mark Harris, Alain Kohl, Richard M. Elliott, Andrew Macdonald

**Affiliations:** 1 Institute of Molecular and Cellular Biology, Faculty of Biological Sciences, University of Leeds, Leeds, United Kingdom; 2 The Roslin Institute, Royal (Dick) School of Veterinary Studies, College of Medicine & Veterinary Medicine, Summerhall, Edinburgh, United Kingdom; 3 Centre for Biomolecular Sciences, University of St. Andrews, Fife, United Kingdom; The Fox Chase Cancer Center, United States of America

## Abstract

The innate immune response provides a critical defense against microbial infections, including viruses. These are recognised by pattern recognition receptors including Toll-like receptors (TLRs) and RIG-I like helicases (RLHs). Detection of virus triggers signalling cascades that induce transcription of type I interferons including IFNβ, which are pivotal for the initiation of an anti-viral state. Despite the essential role of IFNβ in the anti-viral response, there is an incomplete understanding of the negative regulation of IFNβ induction. Here we provide evidence that expression of the Nemo-related protein, optineurin (NRP/FIP2), has a role in the inhibition of virus-triggered IFNβ induction. Over-expression of optineurin inhibited Sendai-virus (SeV) and dsRNA triggered induction of IFNβ, whereas depletion of optineurin with siRNA promoted virus-induced IFNβ production and decreased RNA virus replication. Immunoprecipitation and immunofluorescence studies identified optineurin in a protein complex containing the antiviral protein kinase TBK1 and the ubiquitin ligase TRAF3. Furthermore, mutagenesis studies determined that binding of ubiquitin was essential for both the correct sub-cellular localisation and the inhibitory function of optineurin. This work identifies optineurin as a critical regulator of antiviral signalling and potential target for future antiviral therapy.

## Author Summary

Viral infection stimulates the innate immune response to produce various cytokines and chemokines to induce an anti-viral state within the host. The best studied of these are the type I interferons (IFNα/β), which are essential for an effective anti-viral response. Our understanding of how IFNβ is regulated is not well understood. This study demonstrates that the Nemo-related protein optineurin helps to regulate the levels of IFNβ in response to virus infection. We expressed optineurin in cells and found that the cells failed to express IFNβ when infected with various RNA viruses. Using biochemical experiments we showed that optineurin interacts with the protein kinase TBK1 and the ubiquitin ligase TRAF3. Furthermore, a mutation in optineurin that prevents the interaction with the small protein modifier ubiquitin (D474N) ablated the negative regulatory function of optineurin. Our findings provide a first example of a role for optineurin in anti-viral signalling and aid in our understanding of the negative regulation of IFNβ.

## Introduction

The innate immune response is a highly conserved, first line of defence against microbial pathogens including viruses. To be activated the innate response identifies pathogen associated molecular patterns (PAMPS) [1], which are detected by host pattern recognition receptors (PRRs). The different classes of PRRs implicated in the detection of virus include endosomal Toll-like receptors (TLR), cytosolic DexD/H-box retinoic acid inducible gene-I (RIG-I)-like helicases (RLH) and cytosolic DNA receptors [2] [3] [4] [5],[6],[7]. Activation of PRRs results in the production of pro-inflammatory cytokines and chemokines. Central to this first line of defence are type I interferons (IFNα/IFNβ), which activate transcription of host genes and induce the development of an anti-viral state in the host cell [8]. Loss of IFNβ signalling leads to severe immunodeficiency towards viral infection [9]. All of the anti-viral PRRs can induce the transcription of IFNβ, although the signalling components vary between different PRRs. Common features, however are the recruitment of adaptor proteins (e.g. TLR3 recruits Toll-interleukin 1 receptor domain containing adaptor inducing IFNβ (TRIF)) to form a scaffold, upon which cellular ubiquitin ligases including TRAF3, and the protein kinases TANK-binding kinase 1 (TBK1) and its homologue I-κB kinase (IKKε) are recruited [10].

TBK1/IKKε play a central role during the induction of IFNβ in response to virus infection, underscored by mouse knockout experiments that demonstrate the loss of virus-triggered IFNβ production in TBK1 ^−/−^ mice [11],[12]. Currently, the molecular mechanisms that regulate TBK1/IKKε activation are unclear, although recent studies using the small cell permeant inhibitor BX795 suggest that TBK1/IKKε are phosphorylated on Ser172 by an undetermined protein kinase in response to TLR3 signalling [13]. Phosphorylation of Ser172 leads to an activated form of the kinase that is capable of phosphorylating down-stream substrates including IRF3. Endogenous TBK1/IKKε is found complexed in cells to a number of critical adaptor proteins including TANK [14], NAP1 [15], SINTBAD [16] and the recently described optineurin [17]. These adaptors bind constitutively to TBK1/IKKε and serve to link the kinases to both upstream signalling components as well as down-stream substrates [18] [19]. These studies suggest that there are distinct TBK1/IKKε complexes within the cell that may respond differently to anti-viral signalling [20].

Recently various proteins, including A20, SIKE and RNF125, have been demonstrated to negatively regulate IFNβ induction by targeting TLR/RLH signalling pathways [21],[22],[23]. The TBK1 adaptor protein optineurin (also called NRP (Nemo related protein) and FIP2 (14.7K-interacting protein 2)) inhibits TNFα mediated activation of NFκB by competing with Nemo for binding to RIP1 and is implicated in TNFα induced cell death [24],[25]. Here we describe optineurin as a negative regulator of virus-induced IFNβ induction. Optineurin achieves this via an ubiquitin-dependent protein interaction with TBK1, with which it co-localises within the cell. Over-expression of optineurin inhibits IFNβ expression, thereby increasing viral titres, whereas optineurin siRNA dramatically enhances the IFN-mediated suppression of virus replication. As such we propose that optineurin may represent a broad ranging inhibitor of pro-inflammatory signalling.

## Results

### Expression of optineurin is induced in response to virus infection

**Figure 1:**
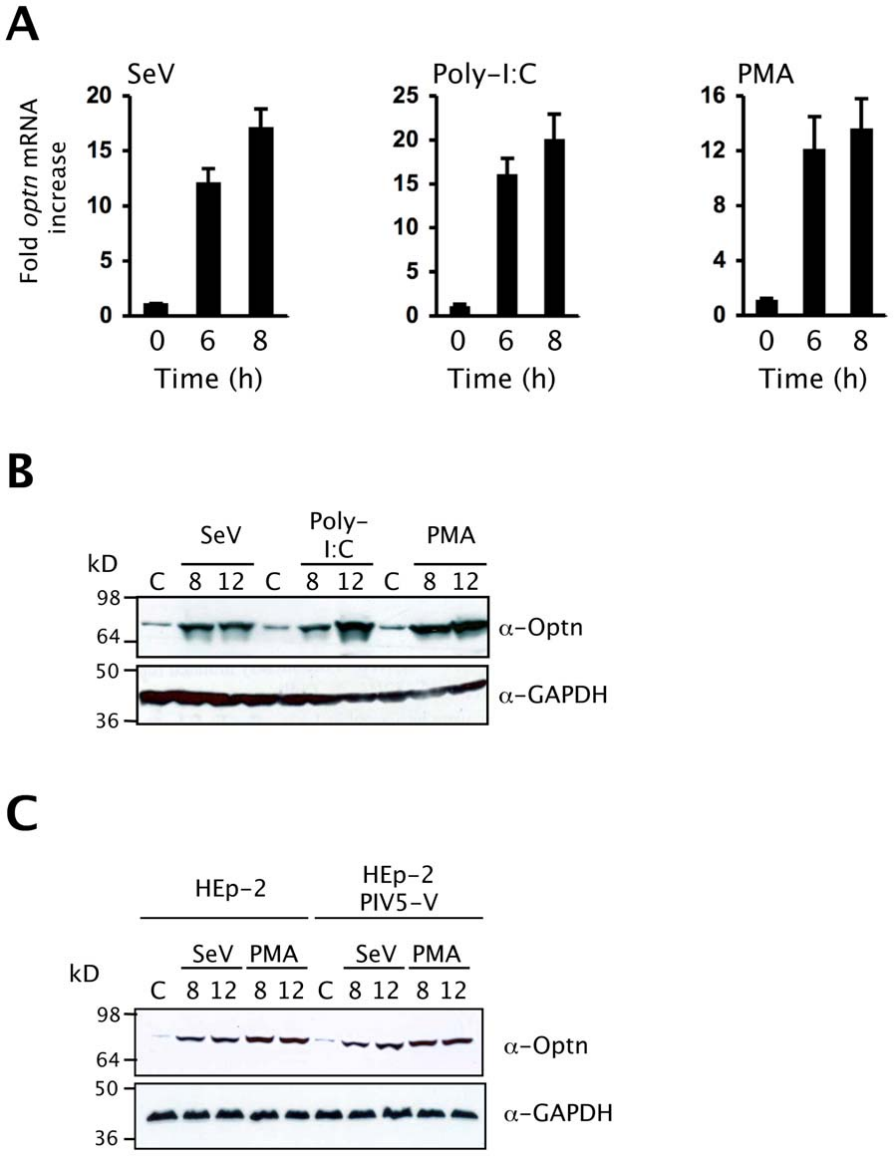
Optineurin expression is induced by virus infection. (A) Serum starved cells were infected with 100 HA units of Sendai virus (SeV), 100 µg/ml poly-I:C or PMA treated (100 ng/ml) and RNA was isolated from cells at the times shown and *optineurin* mRNA levels determined by Q-PCR. Results were corrected for expression of an 18S loading control and calculated relative to an un-stimulated control. Error bars represent the SEM values of stimulations from three independent experiments. (B) Immunoblot of cell lysates 8 and 12 hours after SeV infection (100 HA units/ml), poly-I:C treatment (100 µg/ml) or treatment with PMA (100 ng/ml) analysed with polyclonal anti-optineurin antisera or anti-GAPDH (loading control). All data are representative of at least three independent experiments. (C) Immunoblot of Hep-2 or Hep2-PIV5-V cell lysates 8 and 12 hours after SeV infection (100 HA units/ml) or treatment with PMA (100 ng/ml) analysed with a polyclonal anti-optineurin antisera or anti-GAPDH (loading control).

To investigate whether the expression of optineurin is activated in response to viral infection, HEK293 cells were infected with the RIG-I agonist Sendai virus (SeV) and HEK-TLR3 cells were treated with the TLR3 ligand dsRNA (poly-I:C) (Figure 1). Using qPCR we found that the abundance of optineurin RNA was increased after 6 hours of SeV infection or dsRNA treatment (Figure 1A). Consistent with this we observed increased optineurin protein levels that reached a peak after 12 hours (Figure 1B). Cells that were stimulated with the mitogenic phorbol ester PMA (previously documented to induce the expression of optineurin) [26] demonstrated similar levels of optineurin induction (Figure 1A-B). To confirm that optineurin protein synthesis was directly activated by virus infection and not indirectly through a response to IFN, we repeated the SeV infections in cells engineered to constitutively express a functional V protein of Parainfluenza virus-5 (PIV5) that blocks IFN signaling (Hep2/PIV5-V cells) (Figure S1A) [27],[28] and in Vero cells, which lack the genes for type I interferon [29]. Similar levels of optineurin protein expression were observed in response to SeV infection or PMA treatment in naïve cells, PIV5-V expressing cells and Vero cells, despite the inability of these cells to respond to IFN (Figures 1C and S1B). These data collectively indicate that optineurin expression is increased in direct response to virus infection.

### Over-expression of optineurin inhibits TLR3 and RLH-triggered induction of the IFNβ promoter

**Figure 2:**
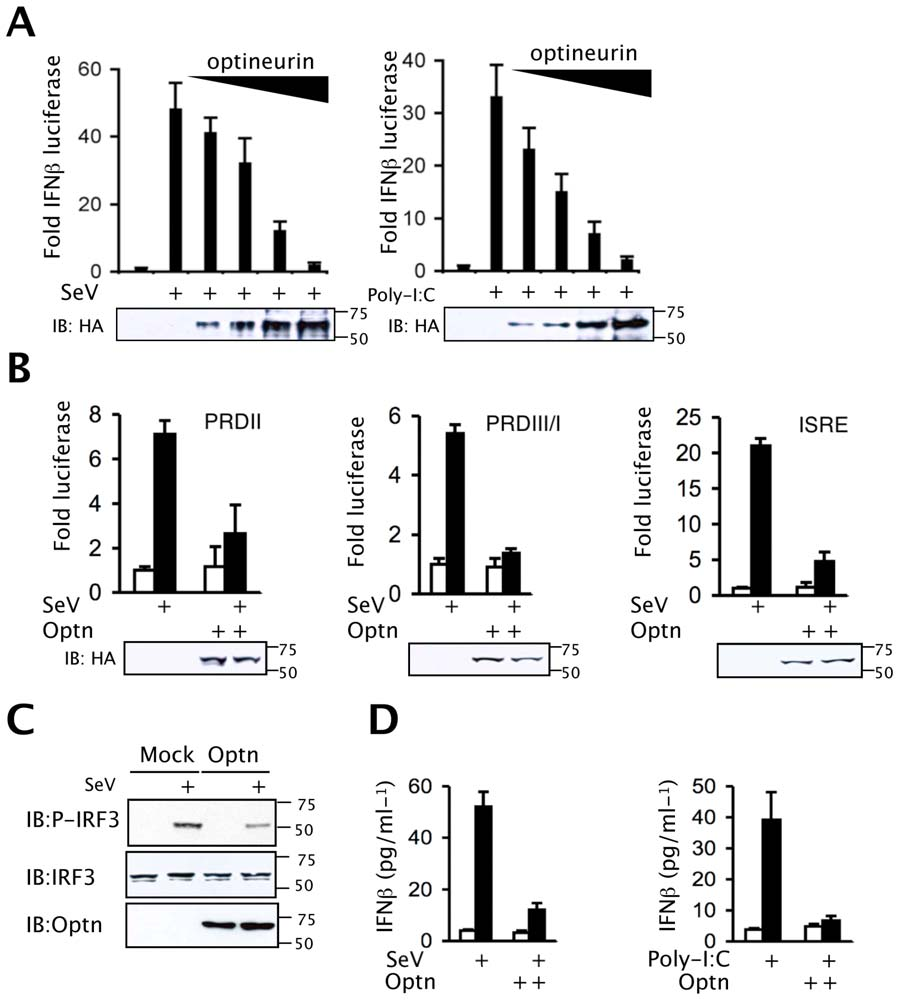
Optineurin inhibits TLR and RLH induction of IFNβ. (A) Effect of optineurin on an IFNβ promoter driven luciferase reporter (pIFNβ-luc) construct. HEK293 or HEK-TLR3 cells were transfected with the IFNβ promoter-driven reporter in the presence of increasing amounts of optineurin expression vector and infected with SeV (100 HA units/ml) (left) or stimulated with extracellular poly-I:C (100 µg/ml) (right). Cells were lysed after 16 hours treatment and analysed for levels of luciferase. (B) HEK293 cells were transfected with either the PRDII, PRDIII/I or ISRE (from ISG54) reporter constructs in combination with optineurin or an empty expression plasmid and infected with SeV (100 HA units/ml). Cells were lysed after 16 hours treatment and analysed for levels of luciferase. Data for all experiments are presented as fold luciferase from an un-stimulated control lacking optineurin co-expression. Error bars are SEM from three independent experiments. (C) HEK293 cells stably expressing optineurin or an empty plasmid control were infected with SeV (100 HA units/ml) and lysed after 8 hours infection. Lysates were analysed for P-IRF3 (Ser396), IRF3, and optineurin by immunoblot. (D) HEK293/HEK-TLR3 cells expressing optineurin or empty plasmid control were infected with SeV (100 HA units/ml) or treated with poly-I:C (100 µg/ml) for 24 hours and IFNβ production was determined by ELISA. Error bars are SEM from three independent experiments.

Since optineurin levels are induced following viral infection we next sought to determine whether optineurin regulates virus-triggered signalling. Using reporter assays, over-expression of optineurin strongly inhibited IFNβ induction in response to SeV infection in a dose-dependent manner (Figure 2A left). Furthermore, over-expression of optineurin also inhibited dsRNA-induced induction of the IFNβ promoter in HEK-TLR3 expressing cells (Figure 2A right). To further define the role of optineurin in the RLH-triggered IFNβ activation pathway, cells were transfected with plasmids encoding RIG-I or Mda-5. Over-expression of RLHs induced a robust activation of the IFNβ reporter, as shown previously [30],[31]. Consistently, optineurin over-expression inhibited activation of the IFNβ promoter in these assays (Figure S2A). Importantly, the observed effects were not due to optineurin-mediated effects on RIG-I or Mda-5 protein expression, as demonstrated by western blotting (Figure S2A). Induction of IFNβ relies on the co-ordinated action of the transcription factors NFκB and IRF3 [32],[33],[34]. In reporter assays optineurin inhibited SeV-triggered activation of the NFκB-dependent PRDII element of the IFNβ promoter (Figure 2B), consistent with previous reports of an optineurin-dependent inhibition of NFκB [24]. Importantly, expression of optineurin inhibited SeV-induced activation of PRDIII/I and ISRE (from the ISG54 gene) reporters, both recognised by activated IRF3 (Figure 2B). In similar experiments optineurin had no inhibitory effects on a serum response element reporter (SRE) or a cAMP response element responsive reporter (CRE) (Figure S2B). In comparison both reporters were successfully inhibited in the presence of known viral antagonists of the MAPK and cAMP pathways, NS5A and NS3, respectively [35],[36],[37].

Western blot analysis revealed that cells expressing optineurin displayed a 60% reduction in IRF3 phosphorylation when infected with SeV and a 50% decrease in IRF3 phosphorylation in response to poly-I:C treatment (Figures 2C and S2C). Blotting with phosphorylation state-independent antibodies showed similar levels of total IRF3 (Figure 2C). These data imply that in cells overexpressing optineurin the phosphorylation of IRF3 and its subsequent activity are inhibited.

Consistent with the promoter reporter assays SeV-induced IFN-β protein levels were also reduced in HEK293 cells over-expressing optineurin (Figure 2D). Taken together, these data indicate that optineurin acts specifically as a negative regulator of the IFNβ response to RNA-activated antiviral signalling pathways.

### Sub cellular localisation of endogenous optineurin

**Figure 3:**
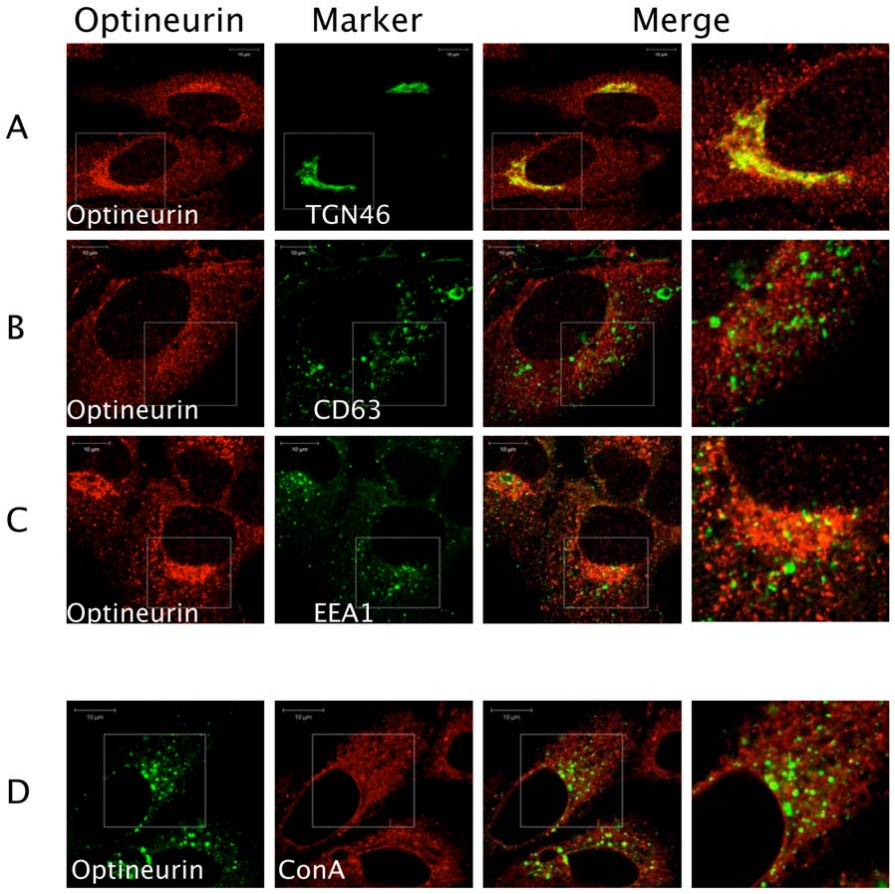
Optineurin distributes to a TGN like cellular compartment. HEK293 cells were serum starved overnight and the following day fixed with ice-cold methanol and permeabilised with 50% methanol/acetone. Optineurin was visualised via labelling with polyclonal rabbit anti-optineurin antibodies followed by staining with antibodies against (A) TGN46, (B) CD63 and (C) EEA1 followed by labelling with Alexa-fluor 488 conjugated secondary antibodies. Labelled ConA (Alexa-594) (D) was added to cells for 1 hour after optineurin labelling with Alexa fluor 488 anti-rabbit secondary antibodies. The indicated images on the right side indicate a higher magnification of the boxed areas. Representative confocal images are shown. Scale bar indicates 10 µM.

Previous studies have shown that optineurin associates with myosin VI and Rab8 [38],[39]. These proteins are involved in the transport of vesicles and cargo recruitment. It was plausible that optineurin might regulate innate immune signalling in the endocytic pathway. We therefore investigated the localisation of endogenous optineurin. Consistent with previous findings optineurin was found to localise to a Golgi-associated compartment as demonstrated by co-staining for TGN46 (Figure 3) [38]. However, a distinct portion of optineurin localised to a broader cytoplasmic region of the cell (Figure 3A), which did not significantly localise with EEA1 or CD63, markers of early and late endosomes respectively (Figure 3B and 3C). In addition, we saw no significant co-localisation of optineurin with concanavalin A (ConA) (Figure 3D), which suggests that optineurin is not localised to the endoplasmic reticulum (ER). When optineurin was transiently over-expressed compared to the endogenous distribution we observed increased accumulation of optineurin in TGN46 positive vesicles, suggesting that over-expressing the protein increases optineurin translocation to this compartment (see below).

### Optineurin interacts with TBK1

**Figure 4:**
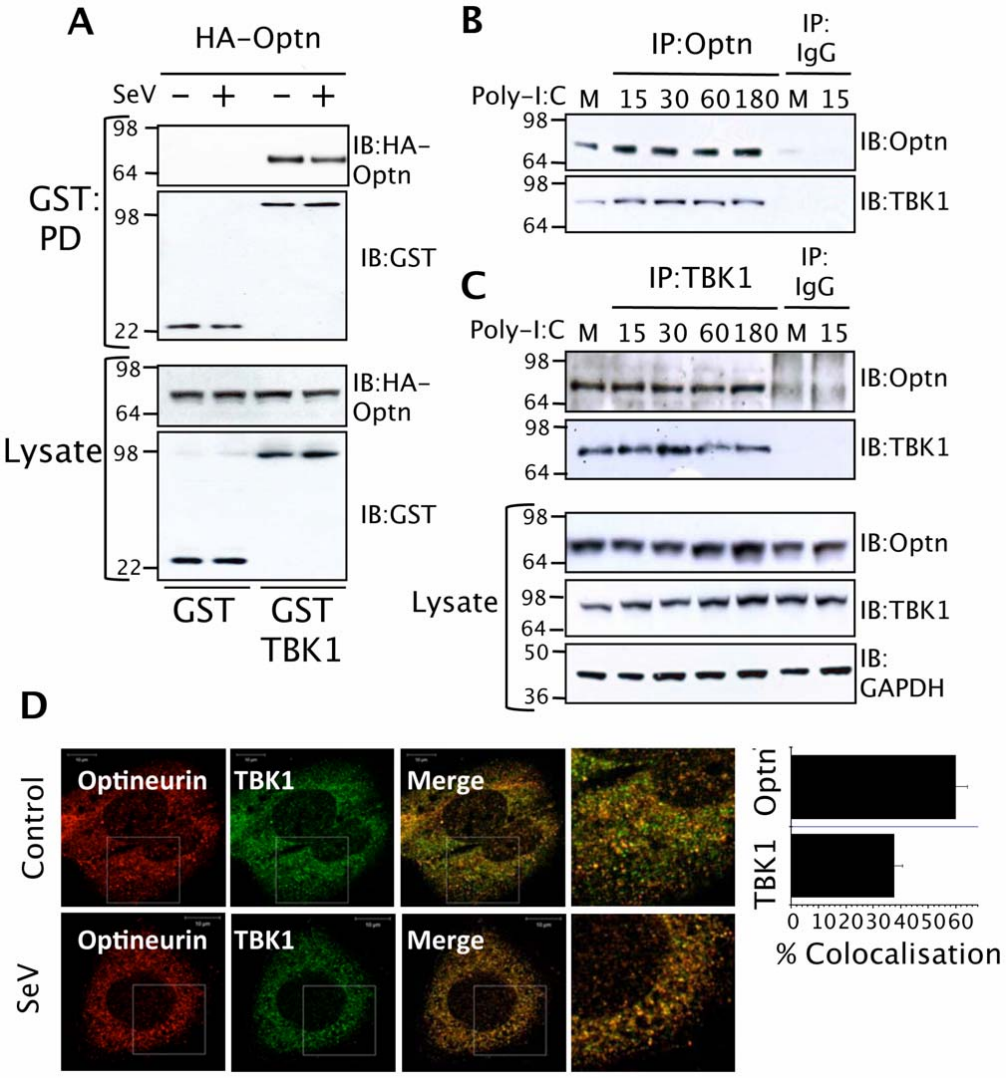
Optineurin interacts with TBK1. (A) HEK293T cells were transfected with HA-tagged optineurin in combination with GST or GST-TBK1. Cells were incubated for 24 hours then infected with SeV (100 HA units/ml) for a further 8 hours before lysis. GST fusion proteins were precipitated from lysates with glutathione-agarose beads and bound optineurin detected by immunoblot analysis. Precipitation of the appropriate GST protein was verified by blotting the precipitates with anti-GST antisera. Expression of all proteins was verified by probing the lysates with anti-HA and anti-GST (+/− denote infection with SeV. PD and IB denote GST pulldown and immunoblot respectively). (B and C) Interaction of endogenous optineurin and TBK1 from RAW264.7 cells. Cells were treated with poly-I:C (100 µg/ml) for the times indicated. Lysates were subjected to immunoprecipitation (IP) with anti-optineurin, anti-TBK1 antibodies or pre-immune IgG coupled to protein G agarose. Immunoprecipitates were immunoblotted with the indicated antibodies. Western blots of the lysates confirmed equal expression of the indicated proteins and an anti-GAPDH blot demonstrated equal loading of protein. (D) Co-immunofluorescence of optineurin and TBK1 showing staining for endogenous optineurin (polyclonal antisera) and endogenous TBK1 (mouse monoclonal antibody). The images were merged to assess co-localisation and the images on the right side indicate a higher magnification of the boxed areas. For quantification of co localization, images were captured as single optical sections of 50 µM thickness and analyzed using IMARIS software using CoLoc and surpass modules. The number of co localized vesicles was expressed as a percentage of the total vesicle count for each of the 488 nm and 594 nm channels using the surpass statistics tab. Each experiment represents the quantified co localization from 20 cells.

We screened likely optineurin-binding partners from the innate anti-viral signalling pathways for an interaction with optineurin by expressing GST-tagged versions of the bait proteins and a HA-tagged optineurin. This successfully detected a constitutive interaction with the protein kinase TBK1, occurring both in the presence and absence of SeV infection (Figure 4A). These experiments were repeated using dsRNA treatment of HEK293-TLR3 cells to activate TLR3 signalling and a similar constitutive interaction between TBK1 and optineurin was detected (data not shown). We next immunoprecipitated endogenous optineurin from a murine macrophage cell line stimulated with poly-I:C and investigated whether it was capable of interacting with TBK1. The optineurin precipitates contained detectable levels of constitutively bound TBK1 (Figure 4B). Additionally, an anti-TBK1 antibody successfully immunoprecipitated optineurin from the same lysates (Figure 4C). In parallel experiments, a control immunoprecipitation undertaken with a pre-immune antibody, failed to precipitate significant levels of TBK1 or optineurin (Figure 4B and C).

To further assess the interaction of TBK1 and optineurin we investigated their intracellular localisation in HEK293 cells in response to SeV infection. We observed prior to stimulation the majority of optineurin localised with TBK-1 and both the binding and amount of optineurin-TBK-1 co-localisation did not change following stimulation, consistent with a constitutive interaction (Figure 4D). The majority of optineurin (∼65%) localised to TBK-1 positive compartments whilst a smaller proportion of total TBK1 was optineurin-associated (Figure 4D).

### Optineurin is found in a complex containing the ubiquitin ligase TRAF3

**Figure 5:**
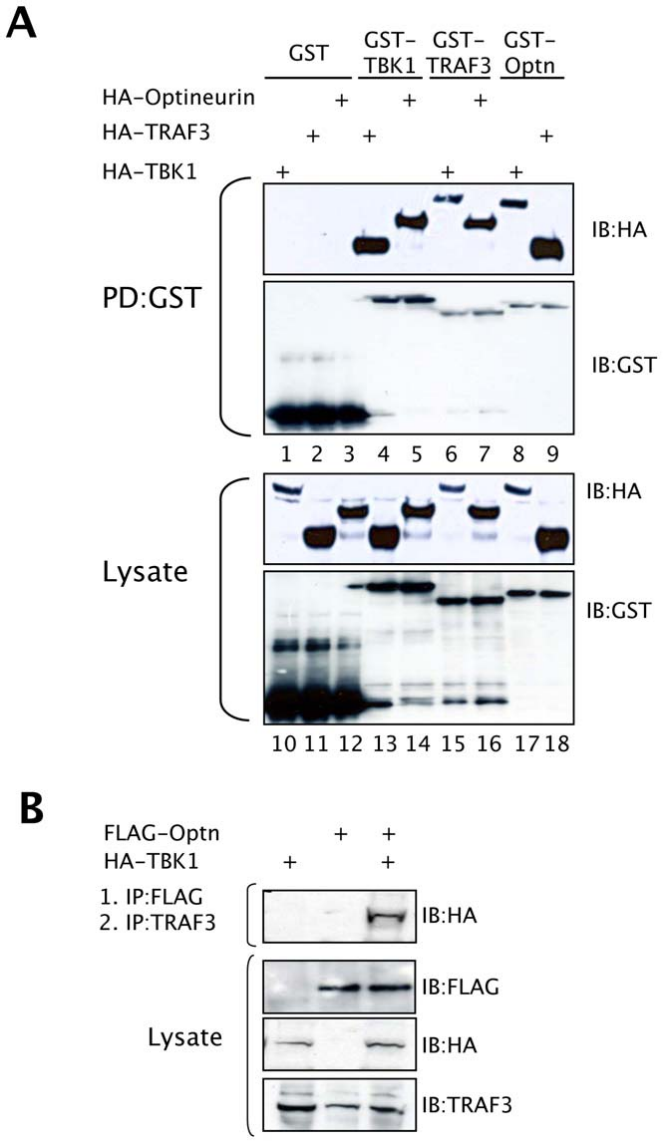
Optineurin is associated with TBK1 and TRAF3. (A) HEK293T cells were transfected with combinations of GST, GST-TBK1, GST-TRAF3 and GST-optineurin and vectors expressing HA-tagged versions of these proteins. After 36 hours cells were lysed and GST pulldowns performed (PD defines pulldown), followed by immunoblotting with an anti-HA monoclonal antibody. (B) HEK293T cells were transfected with a FLAG-optineurin and HA-TBK1 and lysates were immunoprecipitated with FLAG-agarose beads. Precipitates were eluted with a competing FLAG peptide, which was followed by immunoprecipitation with an anti-TRAF3 antibody. Precipitated samples were subjected to immunoblot analysis with an anti-HA antibody to detect bound TBK1. Lysates were also assessed for expression of the appropriate proteins.

TRAF3 is a critical signalling molecule for IFNβ activation in response to virus infection [40], and is a well established binding partner for the TBK1 adaptor protein TANK, residing in a trimeric complex containing TBK1 [19] [41]. GST pull-down experiments confirmed the interactions between TBK1 and optineurin (Figure 5A lanes 5 and 8), TBK1 and TRAF3 (Figure 5A lanes 4 and 6), and importantly, an interaction was observed between optineurin and TRAF3. This interaction was maintained when TRAF3 or optineurin were fused to GST (Figure 5A lanes 7 and 9) and was specific as GST alone failed to interact with any of the HA-tagged binding partners (Figure 5A lanes 1–3). Furthermore, all constructs expressed to approximately equal levels as judged by western blot analysis of cell lysates (Figure 5A lanes 10–18).

To gain a better insight into the nature of this potential protein complex, cells were transfected with a FLAG-tagged optineurin with or without HA-TBK1 and anti-FLAG immunoprecipitations were performed, as described by Gatot *et al*. [41]. The immunoprecipitations were released from the beads by incubating them with a FLAG peptide, and the released material was immunoprecipitated with antibodies to endogenous TRAF3, followed by an anti-HA western blot analysis, which detected the TBK1 (Figure 5B). These data demonstrate that a ternary complex is formed in cells consisting of optineurin, TBK1 and TRAF3.

### The ubiquitin-binding domain of optineurin is required for inhibition of IFNβ induction

**Figure 6:**
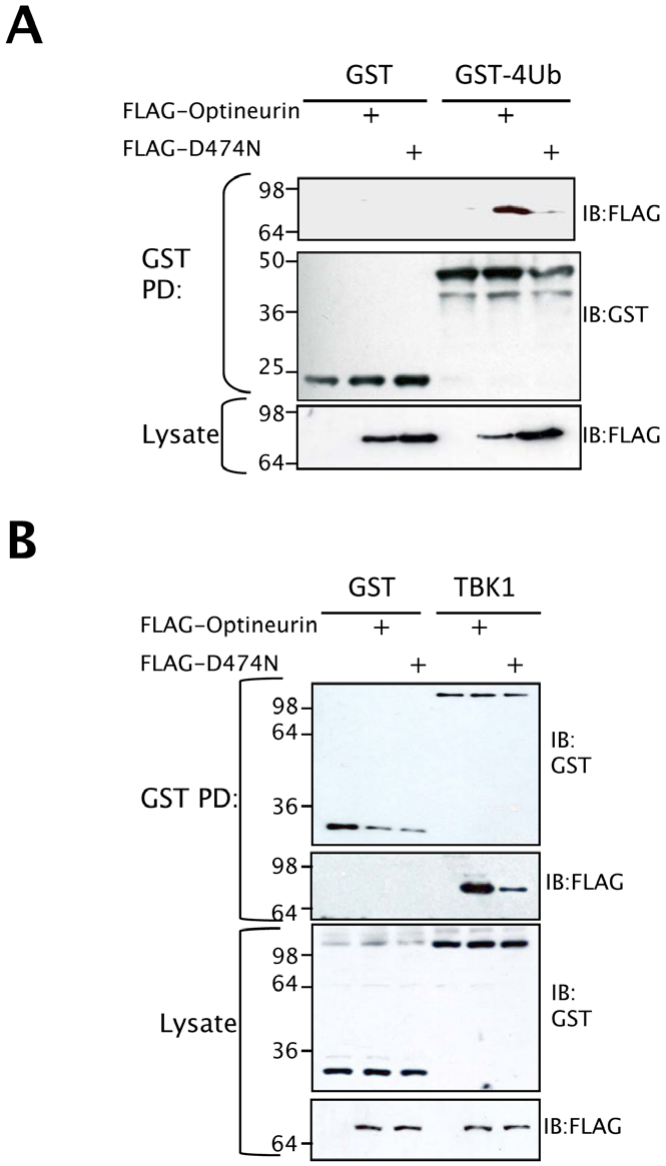
Ubiquitin binding motif of optineurin is required for TBK1 binding. (A) HEK293T cells were transfected with the indicated optineurin plasmids and cell lysates were used for pulldown with bacterial expressed GST or GST-tetraUb (PD denotes pulldown), followed by immunoblotting with an anti-FLAG monoclonal antibody to probe for bound optineurin. (B) HEK293T cells were transfected with GST or GST-TBK1 plus the indicated optineurin plasmid. Cell lysates were precipitated with glutathione agarose beads (PD denotes pulldown). Precipitates were subjected to immunoblotting with monoclonal anti-FLAG antibody to assess binding to optineurin and anti-GST polyclonal antisera to confirm precipitation of the appropriate GST fusion protein. Lysates were probed to demonstrate equal expression of the expressed proteins.

Optineurin was recently identified from a genetic screen as a novel ubiquitin binding protein [42]. Sequence alignment with the related proteins, Nemo and Abin 1–3 revealed that they share a highly conserved DFxxER (Ubiquitin binding in Abin and Nemo (UBAN)) motif that is necessary for ubiquitin binding [42],[43]. To verify the ubiquitin binding abilities of optineurin we created an ubiquitin binding deficient optineurin (D474N) mutant (Figure 6A). The wild type and D474N mutant optineurin were expressed in HEK293T cells and immunoblotting used to verify equal expression and integrity (Figure 6A bottom panels). Bacterial expressed GST tetra-ubiquitin (tetra-Ub) was used to pull-down the optineurin proteins described above from cell lysates. We observed binding of wild-type optineurin to tetra-Ub but the mutation within the UBAN motif reduced binding significantly (Figure 6A).

We reasoned that the ubiquitin-binding motif of optineurin might aid in the interaction with binding partners such as TBK1. To address this issue, the interaction between optineurin and TBK1 was further analysed in cells. Cell lines expressing a GST-tagged TBK1 were co-transfected with FLAG-tagged wild type or D474N optineurin and the TBK1 precipitated with glutathione-agarose beads. GST-TBK1 co-precipitated with wild-type optineurin, whereas GST alone did not (Figure 6B). Interestingly, GST-TBK1 precipitated substantially less optineurin D474N compared to wild-type protein (Figure 6B top panels), with no apparent difference in wild-type and D474N protein expression (Figure 6B bottom panels). These data suggest that the ubiquitin-binding domain (UBAN) of optineurin may therefore be required for the efficient interaction with TBK1.

**Figure 7:**
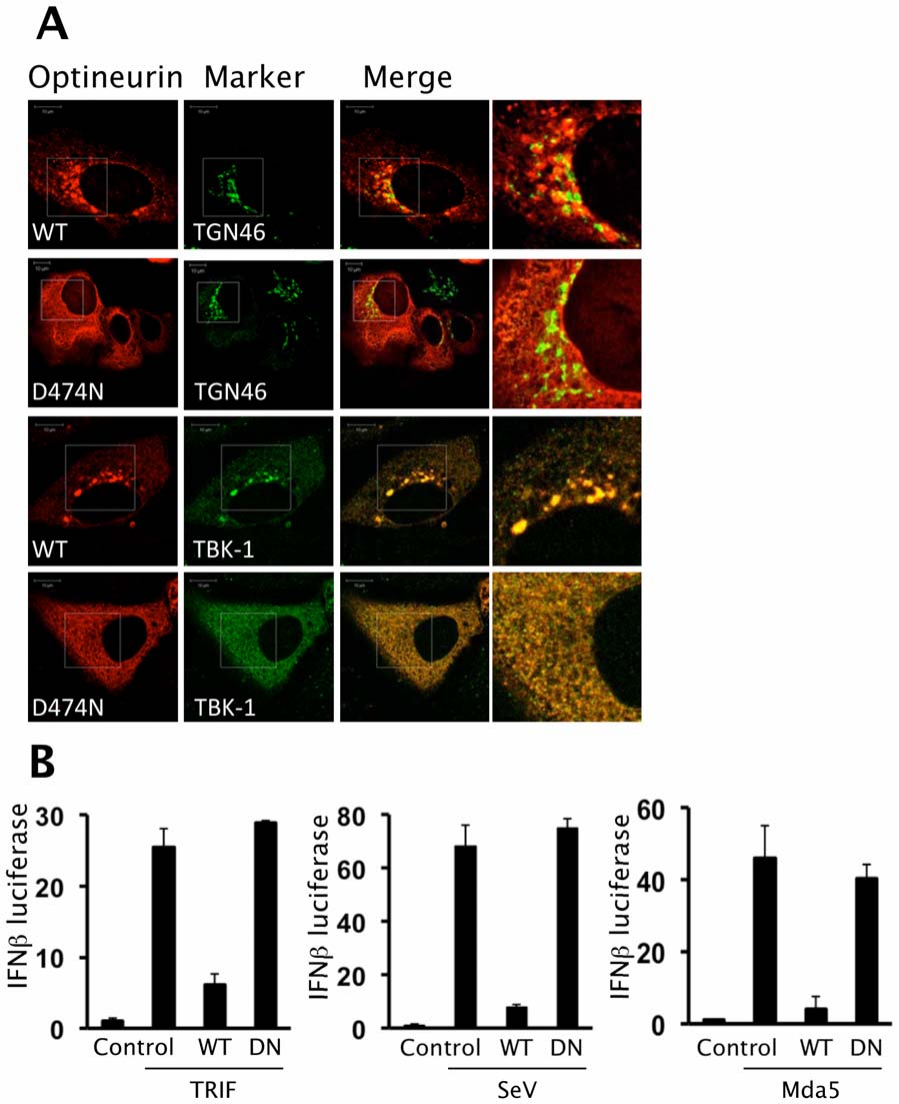
Ubiquitin binding motif of optineurin is required for function. (A) HEK293 cells were transfected with optineurin wildtype and D474N and stained with anti-FLAG antibody to assess the sub-cellular distribution of optineurin. Additionally, cells were stained with markers for the Golgi (TGN46) and endogenous TBK1. The images were merged to assess co-localisation and the images on the right side indicate a higher magnification of the boxed areas. Representative confocal images are shown. Scale bar indicates 10 µM. (B) HEK293 cells were transfected with pIFNβ-luc, optineurin wild-type or D474N and either TRIF or Mda5. The next day cells were left untreated or were infected with 100HA units/ml of SeV for 16 hours. Luciferase levels were determined and are presented as fold luciferase from an un-stimulated control lacking optineurin co-expression. Error bars are SEM from three independent experiments.

When the sub-cellular distribution of the D474N mutant protein was analysed we observed a dramatic redistribution into a cytosolic-like staining pattern whilst the wild-type optineurin clustered around TGN46 positive vesicles (Figure 7A). This suggests that part of the requirement for optineurin to target to these membranes is fulfilled by an interaction with ubiquitin. Consistent with this we observed a high degree of co-localisation of ubiquitin at these clustered sites of optineurin localisation (data not shown). Additionally, endogenous TBK1 displayed co-localisation with over-expressed wild-type optineurin to these large vesicles, whilst the endogenous TBK1 distribution in D474N over-expressing cells remained diffuse (Figure 7A lower panels).

To elucidate the requirement for ubiquitin binding and TBK1 co-localisation on the inhibition of IFNβ we analysed the effect of the optineurin ubiquitin binding deficient mutant (D474N) on activation of IFNβ. In these studies TRIF was expressed ectopically in lieu of extracellular poly-I:C as a means to activate TLR3 signalling. In these reporter assays the optineurin D474N mutant was unable to inhibit the activation of the IFNβ promoter in response to TLR3 or RIG-I signalling (Figure 7B).

### Depletion of optineurin enhances the induction of *ifnβ*

**Figure 8:**
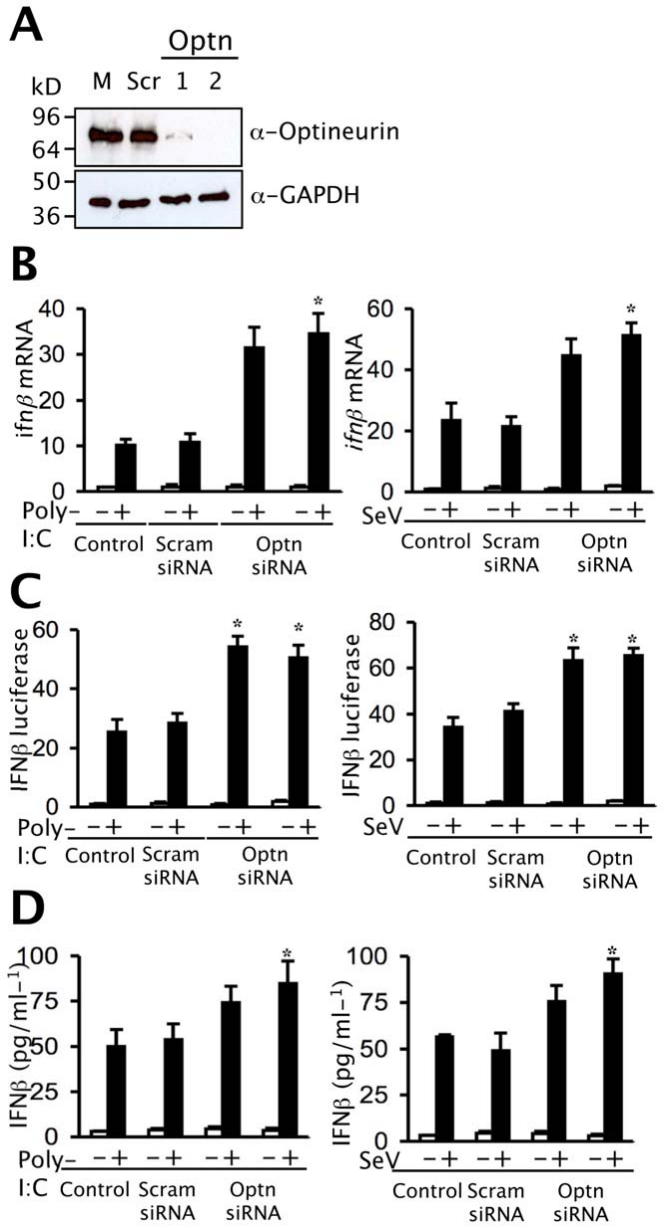
Depletion of optineurin enhances the induction of *ifnβ*. (A) Optineurin levels were reduced by transfection of siRNA oligonucleotides (optn1 and optn2) as demonstrated by immunoblotting with polyclonal anti-optineurin antisera. The siRNA had minimal impact on levels of GAPDH as judged by immunoblotting. (B) Cells treated with optineurin siRNA or control siRNA were infected with 100 HA units/ml SeV or treated with extracellular poly-I:C (100 µg/ml) and RNA was isolated from cells at the times shown and *ifnβ* mRNA levels determined by Q-PCR. Results were corrected for expression of 18S and calculated relative to an un-stimulated control. Error bars represent the SEM values of stimulations from three independent experiments. (C) Levels of IFNβ promoter driven luciferase were determined in optineurin-silenced cells. Cells expressing IFNβ-luc were stimulated as described in (B) and levels of luciferase assayed. Results are shown as fold luciferase from an un-stimulated control. (D) To detect levels of IFNβ protein cells were stimulated as described in (B) and (C) and secreted cytokine was detected by ELISA. Error bars are SEM from three independent experiments. Student's t-test was used and for all tests, a P value of less than 0.05 was considered statistically significant.

To confirm that endogenous optineurin also repressed IFNβ signalling, optineurin specific siRNA oligonucleotides were employed. Immunoblotting demonstrated that two independent optineurin siRNA oligonucleotides (optn1 and optn2) successfully reduced optineurin expression by at least 70% (Figure 8A top), whilst a scrambled control had negligible effect (Figure 8A top). None of the oligonucleotides had a significant effect on the levels of the housekeeping protein GAPDH (Figure 8A bottom). As expected levels of *ifnβ* mRNA rose sharply in response to infection with SeV or treatment with dsRNA (Figure 8B). Silencing of optineurin had no effect on the basal levels of *ifnβ* mRNA in un-stimulated cells but enhanced the subsequent induction of *ifnβ* transcripts by SeV and dsRNA (Figure 8B), whereas the scrambled control oligonucleotide had no effect. Similarly, reporter gene assays revealed that transcriptional activation of the IFNβ promoter in response to SeV and dsRNA was enhanced by optineurin specific siRNA oligonucleotides but not the scrambled control (Figure 8C). Parallel studies utilising the PRDII and ISRE reporter constructs showed increased levels of NFκB and IRF3 driven transcription in optineurin silenced cells infected with SeV (Figure S3). Consistent with the transcriptional data, SeV and dsRNA induced IFN-β protein levels were also greater in the cells transfected with optineurin specific siRNA oligonucleotides (Figure 8D).

### Optineurin regulates the response to virus infection

**Figure 9:**
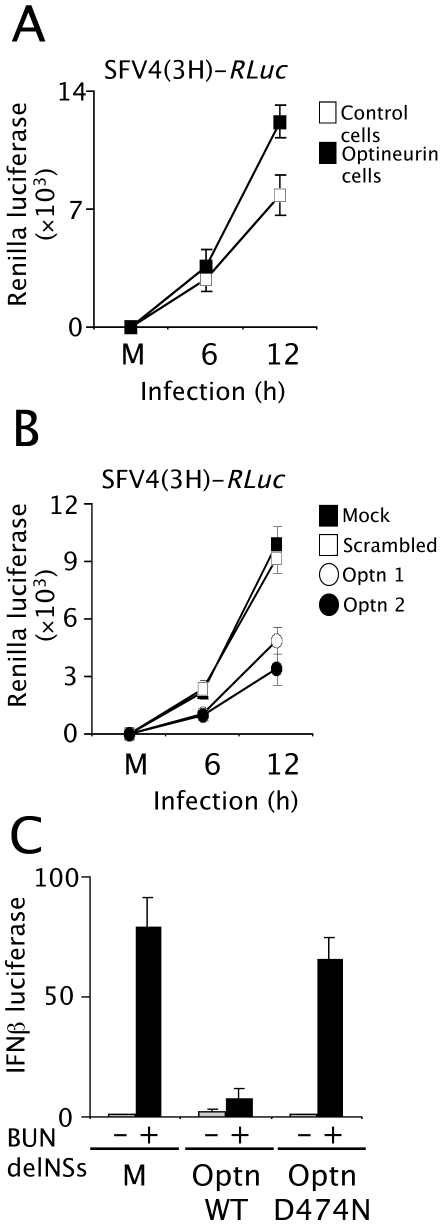
Optineurin regulates the response to virus infection. (A) Cell lines expressing optineurin or empty expression plasmid were infected (MOI 5) with recombinant SFV (SFV4(3H)-*RLuc*) containing a *Renilla* luciferase (*RLuc*) reporter gene. Cells were lysed 8 hours post infection and luciferase levels determined. Data is presented as fold luciferase from uninfected control. Cells expressing optineurin (black boxes), cells expressing empty plasmid (white boxes). (B) Cells treated with optineurin siRNA optn1 and optn2 (black and white circles), control siRNA (white box) or mock treated cells (black box) were infected (MOI 5) with SFV4(3H)-*RLuc*. Cells were lysed 8 hours post infection and luciferase levels determined. Data is presented as fold luciferase from uninfected control. (C) Cells expressing optineurin wildtype or D474N were infected with a Bunyamweravirus lacking the IFN antagonist NSs protein in conjunction with the pIFNβ-luc reporter plasmid. Levels of viral induced IFNβ-promoter driven luciferase were assayed and are displayed as fold luciferase from an uninfected control. Error bars are SEM from three independent experiments.

These studies were extended to measure the effects of optineurin expression on replication of the alphavirus Semliki, Forest virus (SFV), which is highly sensitive to type I interferons [44]. Cell lines constitutively over-expressing optineurin were infected with a recombinant SFV4 virus - SFV4(3H)-*RLuc-* carrying a *Renilla* luciferase (*RLuc*) marker gene. In this virus, *RLuc* is flanked by duplicated nsP2-protease cleavage sites at the nsP3/4 junction as part of the viral non-structural polyprotein [45],[46]. Virus growth curve assays were performed, and these clearly demonstrated that cells over-expressing optineurin produced higher levels of *Renilla* luciferase compared with those expressing empty plasmid (compare white squares [control] to black squares [optineurin over-expressing] Figure 9A). Additionally, SFV4(3H)-*RLuc* replication was measured in optineurin siRNA treated cells. Optineurin siRNA treated cells were more resistant to SFV infection and subsequently produced less luciferase than those treated with the scrambled control siRNA (compare squares [control] to circles [optineurin siRNA] Figure 9B).

As further verification of the role of optineurin in the anti-viral response we analysed the effect of modulating the levels of optineurin on the replication of Bunyamwera virus lacking the IFN antagonist, NSs. Bunyamwera virus is a segmented negative stranded RNA virus and the prototypic member of the *Bunyaviridae* family. The NSs protein acts as a potent IFN antagonist and can effectively block induction of IFNβ [47]. Viruses lacking the NSs protein (BUNdelNSs) are strong inducers of IFNβ [47],[48]. Cells expressing wild-type optineurin or optineurin D474N along with the IFNβ reporter were infected with BUNdelNSs virus. Optineurin had an inhibitory effect on IFNβ reporter activation after infection with BUNdelNSs, whereas the D474N mutant that was incapable of binding to ubiquitin did not (Figure 9C).

## Discussion

During infection cytokine signalling must be controlled to prevent unwanted tissue damage. Host cells have therefore developed strategies to regulate the level of cytokines produced in response to infection. Critical to this regulation are an increasing number of negative regulatory proteins. The results presented in this paper establish optineurin as a novel regulator of virus-triggered IFNβ induction. Over-expression of optineurin inhibited both TLR3 and cytoplasmic helicase (RIG-I/Mda-5) triggered activation of the IFNβ promoter and suppressed the ability of a defective Bunyamweravirus to induce IFNβ. Conversely, cells that were depleted of optineurin by treatment with siRNA were more resistant to SeV and SFV infection, and these cells produced more IFNβ upon infection. Previously it has been described that optineurin is induced by inflammatory cytokines including TNFα [49]. We have now established that virus infection also markedly increases the amount of optineurin protein present in the cells directly as optineurin expression in cells unable to respond to IFN was still up-regulated by virus infection. Promoter mapping studies suggest this up-regulation is likely to be mediated by NFκB [50]. Coupled with the observation that optineurin deficiency did not result in induction of IFNβ, but rather augmented the virus-induced transcription of IFNβ, these data indicate that optineurin levels are increased upon virus infection in order for it to contribute to the fine-tuning of the anti-viral response.

Optineurin has recently been characterised as an NFκB-regulated gene product that interacts with the receptor interacting kinase RIP1 in response to TNFα and initiates a negative feedback loop to inhibit NFκB signalling [24],[50]. The inhibition of both TNFα and IFNβ pathways suggests that optineurin functions as a negative feedback regulator of immune signalling. In this context it will be important to determine the response of optineurin deficient mice to virus infection.

To establish a molecular mechanism for the regulatory role of optineurin we demonstrated an interaction with the protein kinase TBK1. TBK1 is a ubiquitously expressed kinase and a critical point of convergence for TLR and RLH-triggered induction of IFNβ, and as such is a likely candidate for regulation. Characterisation of the optineurin-TBK1 interaction suggests that optineurin is a TBK1 adaptor protein. Binding assays indicated that optineurin was constitutively associated with TBK1, akin to known adaptor proteins including TANK-NAP1-SINTBAD [20] and immunofluorescence analysis demonstrated co-localisation of optineurin and TBK1. Interestingly, quantification of the amounts of optineurin co-localised with TBK1 revealed a significant pool of TBK1 not associated with optineurin, which presumably contained TBK1 complexed with other adaptor proteins (TANK-NAP1-SINTBAD). Morton and colleagues recently identified TBK1 as an optineurin binding partner [17]. Importantly, our findings confirm the constitutive interaction data presented in their study. Moreover, they also identified that optineurin and TANK may share a common binding site on TBK1 [17], which supports the theory that there are distinct pools of TBK1 within a cell, each bound by a specific adaptor protein [20]. TBK1 is closely related to IKKε and is thought to have overlapping functions, including inducing transcription of type I IFN. Both TBK1 and IKKε constitutively interact with TANK-NAP1-SINTBAD [20] and these adaptor proteins are thought to be essential for the overlapping functions of these kinases. Surprisingly, binding assays demonstrated that optineurin was not able to interact with IKKε [17]. If this were the case it suggests that optineurin is the first TBK1 specific adaptor protein found to date and that the TBK1-optineurin complex regulates distinct aspects of the response to virus infection.

It is currently unclear exactly how optineurin regulates the induction of IFNβ. A clue comes from mutagenesis studies, which demonstrate that the ubiquitin-binding motif (UBAN) within optineurin is essential for inhibitory function, as a mutation within this motif (D474N) profoundly impaired the ability of optineurin to inhibit the induction of IFNβ. Furthermore, our studies using this mutant highlighted the requirement of this motif for binding to TBK1. Although, the putative TBK1 binding motif within optineurin has been suggested to locate to the amino terminal third of the protein [17], our data clearly demonstrate that significantly less optineurin (D474N) is bound by TBK1 compared to wild-type protein. The disparity in findings prompted us to investigate the effect of the D474N mutant on optineurin localisation. Immunofluorescence analysis showed that the D474N mutant was not targeted to the same sub-cellular localisation as wild-type protein. Clearly, in this case the ubiquitin-binding motif acts as a potent localisation signal. Moreover, in co-localisation experiments the sites of specific optineurin-TBK1 clustering were lost when optineurin was unable to bind to ubiquitin. Collectively these data suggest that optineurin targets TBK1 to specific sites in the cell and that this is dependent on an interaction with ubiquitin. This was reminiscent of the role of Eps15, which uses an ubiquitin-binding motif to correctly orchestrate formation of protein complexes during receptor tyrosine kinase endocytosis [51] and suggests a model where optineurin may bind to specific ubiquitylated targets to orchestrate specific signalling.

Recent studies suggest that key proteins within the antiviral response are ubiquitylated; including RIG-I and IPS-1 [52],[53] and that several cellular regulators target this ubiquitylation including CYLD, DUBA and RNF125 [23],[54],[55]. Indeed, a mechanism proposed by Zhu and colleagues for the regulation of NFκB by optineurin relies on the competitive recruitment of optineurin to polyubiquitylated RIP1 [24]. The impact of the D474N mutant on the ability of optineurin to inhibit IFNβ induction would argue for a similar mechanism in this case. A role for ubiquitin in the function of optineurin is further strengthened by the observation that optineurin can interact with TRAF3. We speculate that TRAF3 may be required for the polyubiquitylation of optineurin binding partners within the innate signalling pathways, although these targets are not known at this stage. Further studies are needed to ascertain the functional significance of the optineurin-TBK1 sub-cellular targeting and the constituents of any multi-protein complexes, in addition to TRAF3 that contain optineurin. Future studies will undoubtedly identify additional ubiquitylated binding partners for optineurin in antiviral signalling pathways.

In conclusion this study expands the role of optineurin, beyond the negative regulation of TNFα signalling, to include the regulation of virus triggered IFNβ induction. Although, more studies are needed to address the molecular mechanisms by which optineurin regulates the antiviral response, we propose that that optineurin may be a broad-spectrum negative regulator of inflammation.

## Materials and Methods

### Materials

Poly-I:C and human embryonic kidney (HEK) cells expressing TLR3 were purchased from Invivogen. Sendai virus (SeV) Cantell strain was obtained from Charles River Laboratories. The optineurin, TBK1, TRAF3 and HA antibodies were obtained from Abcam. IRF3 and phospho-IRF3 (Ser 396) antibodies were from Cell Signalling Technologies. Anti-FLAG monoclonal antibody, FLAG peptide, protein G and Glutathione agarose were from Sigma.

### DNA manipulations

The luciferase reporter plasmids and Mda-5 expression vector have been described previously [56] and were provided by S. Goodbourn (University of London). The NS5A and NS3 expression vectors have been described previously [29] [31]. The plasmid for bacterial expressed GST-Ub was a kind gift from F. Randow (University of Cambridge). An optineurin expression construct was obtained from F. Buss (University of Cambridge) and used as a template for PCR to generate an optineurin sequence that was cloned into pEBG2T for expression as an amino-terminal GST fusion or with an in-frame amino-terminal FLAG or HA tag that were cloned into pcDNA3.1. A plasmid expressing TRIF was a gift from L. O'Neill (Trinity College, Dublin). RIG-I, TBK1, TRAF3 and IRF3 were amplified with *KOD* polymerase and inserted into vectors for expression in mammalian cells. Site directed mutagenesis of optineurin was performed using a Quick-Change kit (Stratagene).

### Cell culture, transfection and cell lysis

All cells lines were cultured in DMEM supplemented with 10% fetal calf serum, 100 IU/ml penicillin, 100 ug/ml streptomycin and 0.1% Normocin (Invivogen). Routine transfections were carried out using PEI (Polysciences Inc.) at 5 µg/µg DNA according to manufacturer's instructions. Cells were lysed in lysis buffer (50 mM Tris-HCl pH7.5, 1 mM EDTA, 1 mM EGTA, 1% Triton-X100, 1 mM Na_3_VO_4_, 50 mM NaF, 5 mM sodium pyrophosphate, 10 mM sodium glycerophosphate, 0.27 M sucrose and 50 mM iodoacetamide) and placed on ice. Cell lysates were clarified by centrifugation for 20 min at 18,000 g.

### GST pulldowns, immunoprecipitations and immunoblotting

For GST pulldowns cells over-expressing the proteins of interest were lysed in lysis buffer and either used immediately or snap frozen in liquid nitrogen prior to storage at -80°C. 1 mg of total cell lysate was incubated with glutathione-agarose beads for 4 hours at 4°C with constant shaking. For immunoprecipitations involving over-expressed proteins in HEK293T cells, ectopically expressed FLAG tagged protein was precipitated with FLAG-agarose beads (Sigma) for 2 h at 4°C with constant shaking. For precipitations of endogenous optineurin, RAW 264.7 and HEK293 cells treated with poly-I:C (100 µg/ml) or infected with SeV (100 HA units/ml) were lysed and 1 mg of total lysate was incubated with 5 µg of anti-optineurin antibody for 2 h at 4°C and then incubated with protein G agarose beads overnight. All precipitates were washed thoroughly in lysis buffer, and proteins released from the beads with the addition of Laemmli loading buffer. Precipitated proteins were analysed by SDS PAGE, transferred to PVDF membrane and immunoblotted. Levels of phosphorylated IRF3 were quantified by Image densitometry imaging to analyze intensity of western blot bands. The signal intensities for quantification were normalized to the background values and this signal subsequently normalized to the levels of total IRF3.

### Immunofluorescence

Cells grown on glass coverslips were transfected with either FLAG-tagged optineurin (for over-expression studies) or the indicated constructs (where applicable). 48 h post-seeding or transfection, cells were fixed with ice-cold methanol for 10 minutes, followed by permeabilisation in ice-cold methanol/acetone for 10 minutes. Cells were washed with PBS and blocked in PBS/1 % BSA for 30 min. Cells were then incubated with a rabbit polyclonal anti-optineurin antibody (Abcam) for 1 h (for investigation of endogenous localisation) or with mouse anti-FLAG antibody (for over-expression studies) in PBS/1 % BSA and washed with PBS prior to incubation with Alexa–Fluor 594 conjugated anti goat (rabbit) or anti-mouse secondary antibody (Invitrogen-Molecular Probes) in PBS/1 % BSA for 1 hour at room temperature. Cells were probed with sheep anti-TGN46 conjugated to anti-sheep 488 antibodies for Golgi investigation, anti-mouse EEA1 or anti-CD63 antibodies conjugated to anti-mouse secondaries for endosomal investigation. Cells were washed and mounted onto microscope slides using Citifluor (Agar Scientific). Labelled cells were viewed on a Zeiss 510-META laser scanning confocal microscope under an oil-immersion ×63 objective lens (NA = 1.40). Alexa-fluor 594, (550 nm excitation: 570 nm emission) was excited using a helium/neon laser fitted with 543 nm filters. Images displayed are representative and displayed as single optical sections of 50 µM thickness.

For quantification of co localization, images were captured as single optical sections of 50 µM thickness (maintaning identical channel settings throughout) and analyzed using IMARIS software using CoLoc and surpass modules. Briefly the thresholds of the each channel were set at 10% of the maximum intensity and vesicles of a diameter of 0.5 µM were recorded in both the 488 nm and 594 nm channels using Imaris to calculate the structures that fall into this sizing. The number of vesicles was then entered into the corresponding channel thresholds in the coLoc module and white pixels appeared on the image to show the location of co-localized pixels. The number of co localized vesicles was then expressed as a percentage of the total vesicle count for each of the 488 nm and 594 nm channels using the surpass statistics tab. Each experiment represents the quantified co localization from 20 cells.

### Reporter assays

Cells (1×10^5^) were seeded into 12 well dishes and transfected the following day using PEI (Polysciences Inc.) with reporter plasmids expressing firefly luciferase under the control of the complete IFNβ promoter, the PRDII, PRDIII/I elements of the IFNβ promoter or a tandem ISRE element taken from the ISG54 promoter. Where appropriate, cells were co-transfected with plasmids expressing cellular proteins (e.g. Mda5). Empty plasmid was added to ensure each transfection received the same amount of total DNA. To normalise for transfection efficiency pRLTK Renilla luciferase reporter plasmid was added to each transfection. Where necessary, 24 hours post transfection cells were treated with 100 µg/ml poly-I:C or infected with 100 HA units/ml Sendai virus for a further 16 hours. Samples were lysed in passive lysis buffer (Promega) and activity measured using a dual-luciferase reporter assay system (Promega) as described [37].

### SFV virus preparation and infection

Recombinant *Renilla* luciferase-expressing SFV4(3H)-*RLuc* (derived from strain SFV4) [46] was grown in BHK-21 cells (37°C; in MEM/2% newborn calf serum (NBCS). Virus-containing supernatants were clarified by centrifugation (3x, 30 minutes, 15000 rpm) and viruses concentrated from supernatant on a 20% (w/v) sucrose/TNE buffer (pH 7.4) cushion by ultracentrifugation (25000 rpm, 90 minutes, SW28 rotor). Pellets were resuspended in TNE buffer, and viruses titrated by plaque assay. Infection of mammalian cells was performed at 37 °C for 1 hour, respectively, at an m.o.i. of 5 plaque forming units (pfu) per cell in DMEM containing 0.5% foetal calf serum. After infection complete medium was added to the cells.

### RNA interference

Decreased optineurin expression was obtained using pre-validated siRNA molecules (Ambion). These were transfected using the siPort Neofect reagent according to the protocol provided by the manufacturer (Ambion).

### Detection of cellular mRNA

Cells were transfected with siRNA to silence endogenous optineurin as described. Cells were stimulated with agonist and incubated for the times indicated. Total RNA was extracted using a Nucleospin kit (Machery-Nagel) and cDNA was generated from 1 µg of total RNA using the Super-Script II reagent (BioRad). The resulting cDNA was subjected to semi-quantitative real time PCR using the SYBR green reagent (BioRad) as previously described [57].

### Determination of IFNβ protein levels

HEK293 cells expressing optineurin or treated with optineurin specific siRNA were infected with SeV (100 HA units/ml) or treated with poly-I:C (100 µg/ml) and levels of secreted IFNβ detected by ELISA using the manufacturer's protocols (PLB Interferon Source).

## Supporting Information

Figure S1 (A) HEp-2 parental and HEp-2 cells expressing the PIV5 V protein (HEp-2/PIV5-V) cells were transfected with luciferase reporter contructs pIFNβ or pISRE and stimulated with SeV infection or 1000 IU/ml IFNα. Expression of PIV5-V protein had no significant effect on the levels of luciferase generated from the pIFNβ reporter, demonstrating that V is not able to inhibit the production of IFN. In contrast expression of V led to a statistically significant decrease in pISRE driven luciferase. These data show that cells expressing PIV5-V are able to produce IFN but not able to respond to it. ** corresponds to a <P0.05. (B) Vero cells were serum starved for 24 hours then infected with SeV (100 HA units/ml) or treated with PMA (100 ng/ml) for the indicated times. Cell lysates were analysed with a polyclonal anti-optineurin antibody and anti-GAPDH to show equal protein loading.
(0.50 MB TIF)

Figure S2 (A) Cells containing pIFNβ-luc were co-transfected with RIG-I (left) or Mda5 (right) expression plasmids in the presence of increasing concentrations of optineurin expression vector. (B) Cells were transfected with a serum responsive element (SRE)-reporter (left) or (C) a cAMP responsive element (CRE) reporter (right) in combination with optineurin or the appropriate controls, HCV NS5A (left) and HCV NS3 (right). Cells were stimulated with serum (SRE) or forskolin (CRE) (right) for 16 hours. Data for all experiments are presented as fold luciferase from an unstimulated control lacking optineurin co-expression. Error bars are SEM from three independent experiments. (D) HEK293 cells stably expressing optineurin or an empty plasmid control were infected with SeV (100 HA units/ml) and lysed after 8 hours infection. Lysates were analysed for P-IRF3 (Ser396), IRF3, and optineurin by immunoblot.
(0.90 MB TIF)

Figure S3 Optineurin siRNA enhances PRDII and ISRE activation. HEK293 expressing (A)PRDII or (B) ISRE reporter constructs were treated with scrambled or optineurin specific siRNA stimulated with SeV (100 HA Units/ml) for 18 hours and levels of luciferase assayed. Results are shown as fold luciferase from an unstimulated control. Error bars are SEM from three independent experiments.
(0.33 MB TIF)
