# Supplementary figures and images for: Optineurin Negatively Regulates the Induction of IFNβ in Response to RNA Virus Infection
Source: PLoS Pathog. 2010 Feb 19;6(2):e1000778. doi: 10.1371/journal.ppat.1000778 (PMC2824764; doi:10.1371/journal.ppat.1000778)

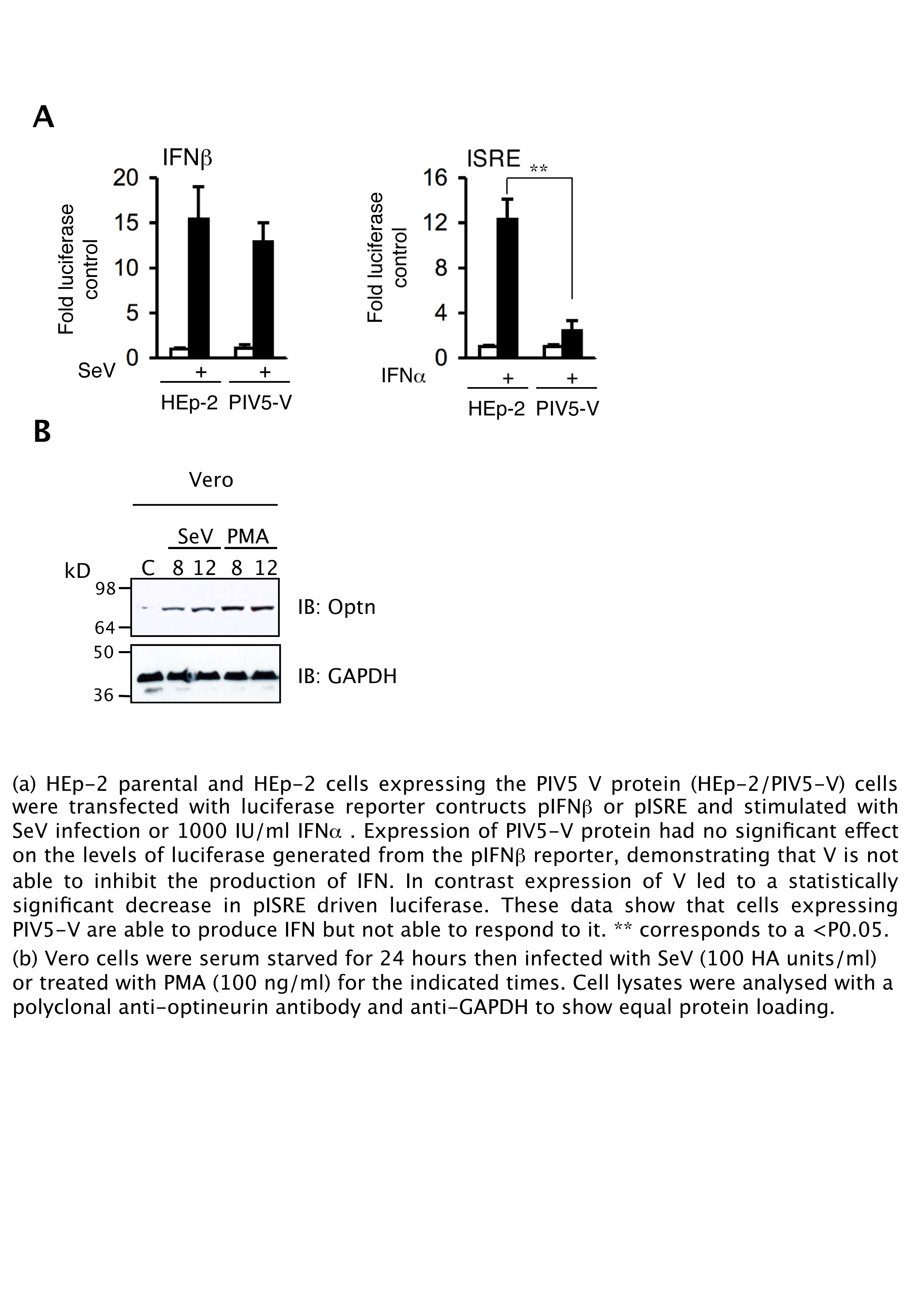

Supplement: Figure S1 — (A) HEp-2 parental and HEp-2 cells expressing the PIV5 V protein (HEp-2/PIV5-V) cells were transfected with luciferase reporter contructs pIFNβ or pISRE and stimulated with SeV infection or 1000 IU/ml IFNα. Expression of PIV5-V protein had no significant effect on the levels of luciferase generated from the pIFNβ reporter, demonstrating that V is not able to inhibit the production of IFN. In contrast expression of V led to a statistically significant decrease in pISRE driven luciferase. These data show that cells expressing PIV5-V are able to produce IFN but not able to respond to it. ** corresponds to a <P0.05. (B) Vero cells were serum starved for 24 hours then infected with SeV (100 HA units/ml) or treated with PMA (100 ng/ml) for the indicated times. Cell lysates were analysed with a polyclonal anti-optineurin antibody and anti-GAPDH to show equal protein loading. (0.50 MB TIF) [file ppat.1000778.s001.tif]

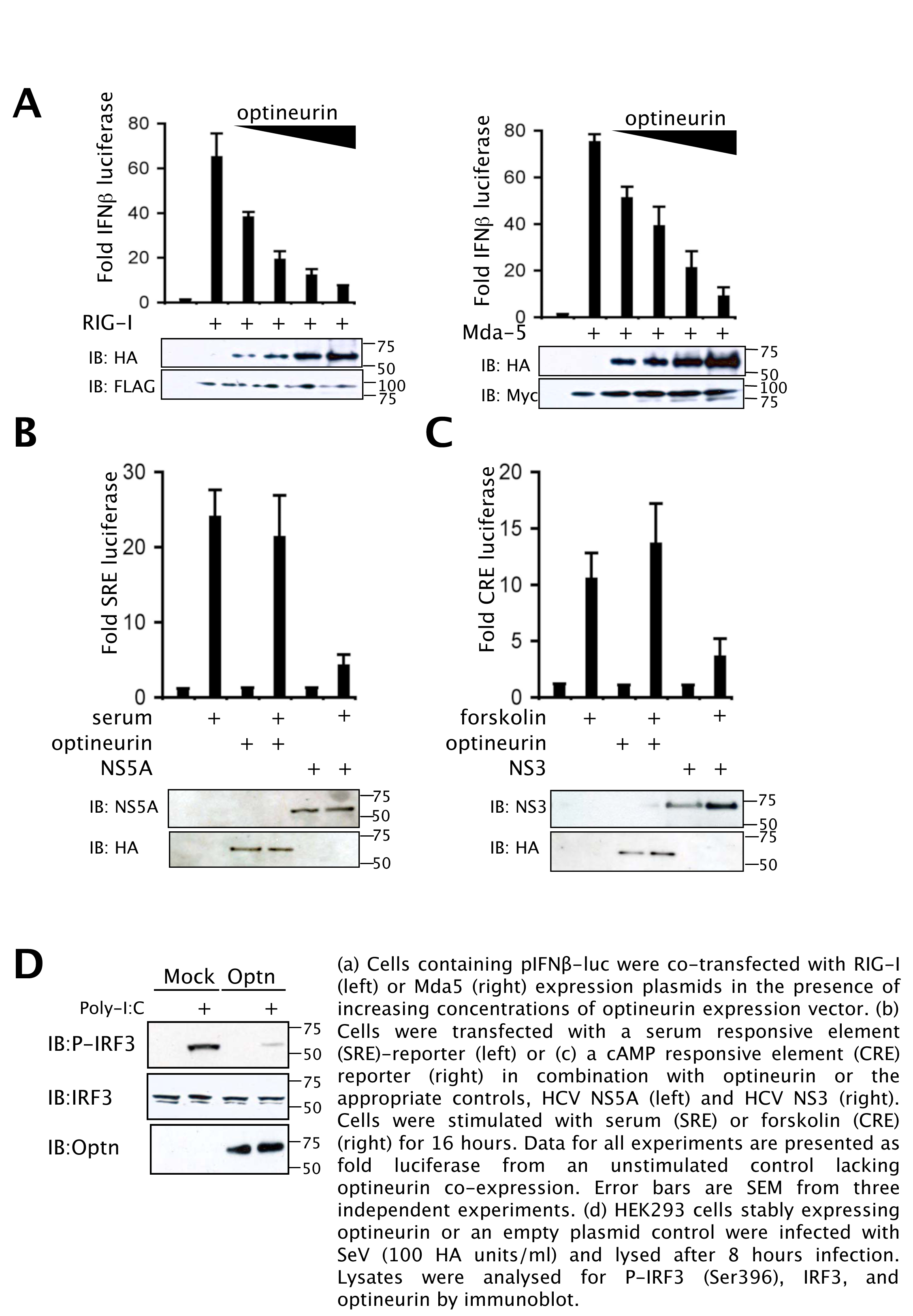

Supplement: Figure S2 — (A) Cells containing pIFNβ-luc were co-transfected with RIG-I (left) or Mda5 (right) expression plasmids in the presence of increasing concentrations of optineurin expression vector. (B) Cells were transfected with a serum responsive element (SRE)-reporter (left) or (C) a cAMP responsive element (CRE) reporter (right) in combination with optineurin or the appropriate controls, HCV NS5A (left) and HCV NS3 (right). Cells were stimulated with serum (SRE) or forskolin (CRE) (right) for 16 hours. Data for all experiments are presented as fold luciferase from an unstimulated control lacking optineurin co-expression. Error bars are SEM from three independent experiments. (D) HEK293 cells stably expressing optineurin or an empty plasmid control were infected with SeV (100 HA units/ml) and lysed after 8 hours infection. Lysates were analysed for P-IRF3 (Ser396), IRF3, and optineurin by immunoblot. (0.90 MB TIF) [file ppat.1000778.s002.tif]

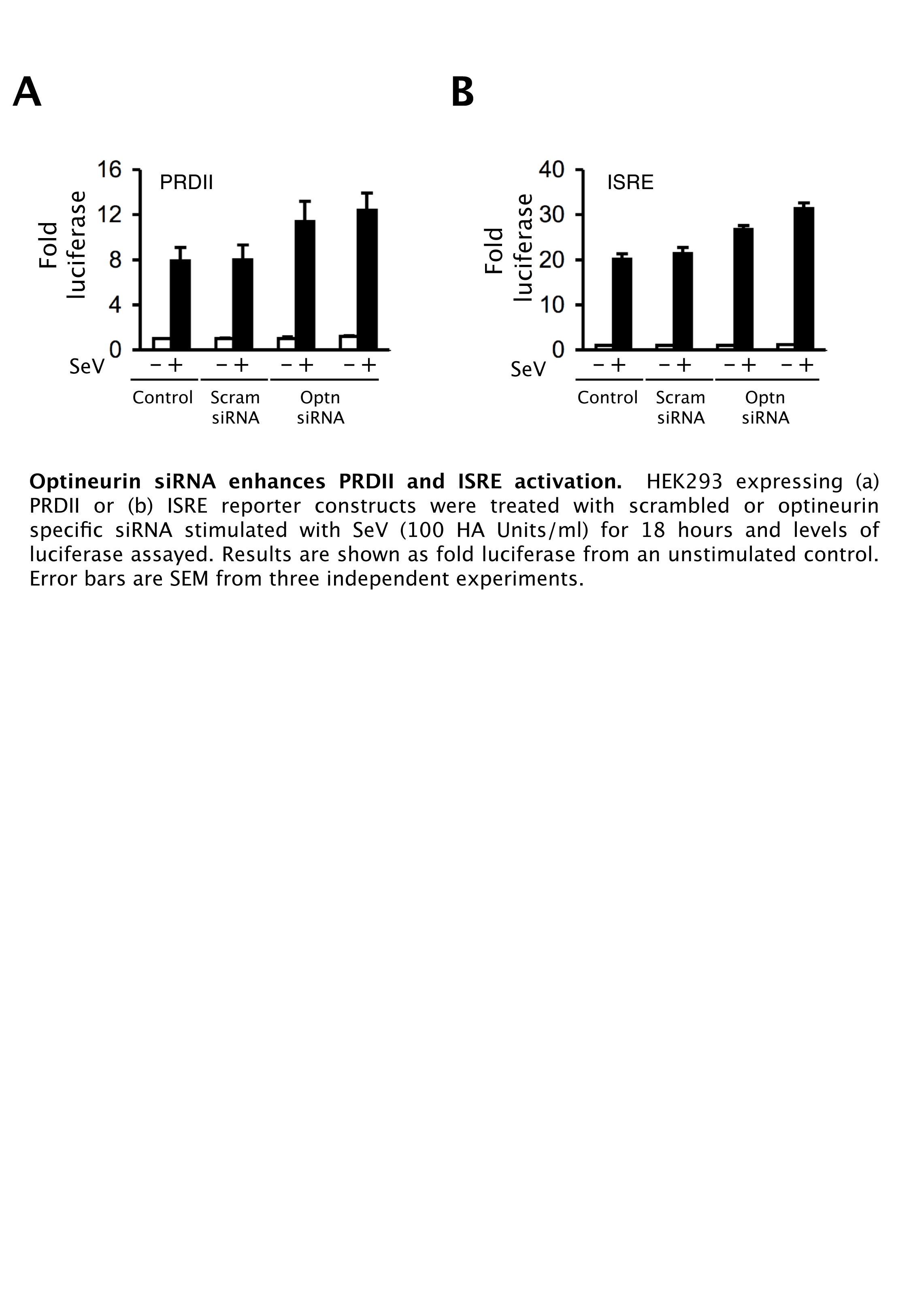

Supplement: Figure S3 — Optineurin siRNA enhances PRDII and ISRE activation. HEK293 expressing (A)PRDII or (B) ISRE reporter constructs were treated with scrambled or optineurin specific siRNA stimulated with SeV (100 HA Units/ml) for 18 hours and levels of luciferase assayed. Results are shown as fold luciferase from an unstimulated control. Error bars are SEM from three independent experiments. (0.33 MB TIF) [file ppat.1000778.s003.tif]
